# Zinc isotope ratios of bones and teeth as new dietary indicators: results from a modern food web (Koobi Fora, Kenya)

**DOI:** 10.1038/srep26281

**Published:** 2016-05-18

**Authors:** Klervia Jaouen, Melanie Beasley, Margaret Schoeninger, Jean-Jacques Hublin, Michael P. Richards

**Affiliations:** 1 Max Planck Institute for Evolutionary Anthropology, Department of Human Evolution, Deutscher Platz 6, 04103, Leipzig, Germany; 2 University of California Department of Anthropology, 9500 Gilman Dr. La Jolla, CA 92093, USA; 3 Department of Archaeology, Simon Fraser University, Burnaby, B.C., Canada, V5A 1S6

## Abstract

In order to explore the possibilities of using zinc (Zn) stable isotope ratios as dietary indicators, we report here on the measurements of the ratio of stable isotopes of zinc (^66^Zn/^64^Zn, expressed here as δ^66^Zn) in bioapatite (bone and dental enamel) of animals from a modern food web in the Koobi Fora region of the Turkana Basin in Kenya. We demonstrate that δ^66^Zn values in both bone and enamel allow a clear distinction between carnivores and herbivores from this food web. Differences were also observed between browsers and grazers as well as between carnivores that consumed bone (i.e. hyenas) compared to those that largely consume flesh (i.e. lions). We conclude that Zn isotope ratio measurements of bone and teeth are a new and promising dietary indicator.

With recent progresses in mass spectrometry, it is now possible to measure precisely and accurately the stable isotope compositions of a range of trace elements in animal tissues (e. g., bone, dental enamel, blood). Preliminary studies demonstrated that the isotopic compositions of these non-traditional elements in animal tissues were related to their diets^1^,^2^,^3^,^4^,^5^,^6^,^7^,^8^,^9^, and in human blood Zn isotope values could be used to detect meat-consumption^10^,^11^. Here we present the largest study to date of Zn isotope measurements of plants and animals from a modern food web from Koobi Fora, Kenya, as well as the first Zn isotopic values for dental enamel. We undertook this study to explore the differences between different members of the food web, and specifically to determine if we could distinguish between herbivores and carnivores using Zn isotope ratios on bone and teeth.

Zinc has five stable isotopes - ^64^Zn, ^66^Zn, ^67^Zn, ^68^Zn, and ^70^Zn - with respective average natural abundances of 48.6, 27.9, 4.1, 18.8, and 0.6%. As the isotopes 64 and 66 are the most abundant, the ratio of ^66^Zn/^64^Zn expressed as the δ^66^Zn value is calculated using these two isotopes. Mass independent fractionation of Zn isotopes have been predicted during Zn redox reactions^12^, but since in animals and plants Zn is only present in the valence state as Zn^2+^, the fractionation within these organisms is mass-dependent^3^,^13^,^14^,^15^. Because of its unique oxidation state in biological organisms, the isotopic fractionation of Zn is expected to occur only during Zn exchanges between ligands^14^,^16^. The total isotopic variation observed in plants and animal tissues usually vary between −1‰ and +1‰^11^,^15^,^17^,^18^.

Two dietary factors are likely to impact body Zn isotopic compositions: the isotopic fractionation occurring during intestinal absorption and the actual Zn isotopic composition of the food products. There have been a small number of studies that have explored the relationship between Zn isotope ratios and diet. A ^66^Zn-enrichment relative to ^64^Zn apparently occurs in body tissues during Zn intestinal absorption from plants, which has been attributed to the precipitation of dietary Zn with phytates in the intestine, inhibiting Zn absorption, and favoring the binding of Zn light isotopes^3^,^13^,^19^. Due to this fractionation during intestinal absorption, the Zn isotopic ratios of herbivore body tissues should be higher than the Zn isotope values of their diets^3^,^13^. Carnivores, which do not consume phytates (which are associated with Zn), should consequently have less of a fractionation between food Zn isotope ratios and their body tissue Zn isotope values. These predictions are consistent with two studies on living humans, which demonstrated that δ^66^Zn values for human blood were lower in omnivores than in vegetarians^10^,^11^.

There are also expected variations in Zn body tissue composition between herbivore grazers and browsers. This is not due to physiological effects, such as observed between carnivores and herbivores, but instead due to the difference in Zn isotope composition of leaves compared to other parts of plants. Leaves are known to be relatively enriched in Zn light isotopes compared to the stem and rhizomes of the same plant, and Viers *et al*.^18^ demonstrated that tree leaves are strongly ^66^Zn-depleted relative to ^64^Zn compared to the different parts of herbaceous species coming from the same soil. Leaf consumption is therefore expected to result in decreased Zn isotopic ratios in herbivore tissues. Therefore, browsers - whose diet relies largely on leaves - should exhibit lower Zn isotope values than grazers, which eat whole plants.

Different body tissues within the same animal may also have different Zn isotope compositions. Two recent controlled feeding studies have shown that for modern mammals, muscle tissue and bone have a different Zn isotopic composition^14^,^20^. These studies showed that muscle tissue actually had ^66^Zn–depleted values compared to the food (Δ^66^Zn_muscles-food_ ≈−0.2‰), while bone had ^66^Zn-enriched values (Δ^66^Zn_bones-food_≈0.1 to 0.4‰). In contrast, a feeding experiment using sheep reported that, for the four animals analyzed, there were similar values between bone and muscle tissue, and both tissues were enriched in heavy isotopes relative to their diet^13^.

The measurements of the stable isotope values of a range of trace elements, including Zn, have been previously reported for two modern food webs in South Africa^3^, where a significant overlap between herbivores and carnivores was observed, albeit with herbivores clearly exhibiting the higher Zn isotopic values. This first study used animal samples from wide geographic ranges in each study area. The two sites had differing underlying rocks, and as plant and animal Zn isotope ratios are likely to be related to the local bedrock and soil Zn isotope values, animals feeding in different bedrock substrates within each study area may not be directly comparable to each other. It seems likely that the differing bedrock compositions could explain the overlap that was observed by Jaouen *et al*.^3^ in herbivore and carnivore values. If so, only animals from single foodwebs in the same geographical region with the same underlying bedrock should be directly compared to each other.

Here, we build upon these earlier studies, and particularly the study of the two South African food webs, to look at Zn isotopes in animals and plants from a single geographical region consuming plants from a single geological substrate (i.e., the Lake Turkana Basin in northern Kenya, Fig. 1). In this region, we should see differences in Zn isotope between herbivores and carnivores, and between browsers and grazers. We argue that such differences would be due solely to physiological and/or dietary differences between species.

We measured the stable isotope ratios of zinc (^66^Zn/^64^Zn) in plants, bones and dental enamel from the modern African food web in the Koobi Fora region in the Turkana Basin of Kenya. Enamel and bone were sampled from the same individuals. Samples consisted of 8 different plant species and teeth and bone from 37 modern animals, including mammals and reptiles (crocodiles) from 12 different species. Of these species, we measured browsers (3 species), grazers (3 species) and carnivores (6 species). We selected samples coming from a small geographical area (radius <15 km) with the exception of three that were collected from other, nearby locations (Fig. 1). The underlying geology is composed of Quaternary sediments and tuffs (volcanic deposits)^21^,^22^. Igneous and clastic rocks have very homogenous Zn isotopic compositions^23^,^24^,^25^,^26^,^27^, so we would expect that each different geological region would have a homogenous Zn isotope value. Isotope variation between the different areas, however, could be related to different amounts of carbonates in the underlying geology, which are known to be enriched in Zn heavy isotopes^25^. A second factor of variation could be due to environmental factors, such as soil weathering and vegetation, which can fractionate soil Zn isotopes within a geological zone^18^,^25^. Nevertheless, because of the semi-arid climate of the Turkana Lake region, Koobi Fora’s soil is sodic^28^, which impedes water infiltration and plant growth. Therefore, the soils in each of the different geological areas studied here will have homogenous isotopic compositions, unless their carbonate content varies. This additional layer of complexity needs to therefore be taken into account when interpreting Zn isotope differences between individuals from the same species, but living in different locations

## Results

An overall characteristic of this dataset is that bones are enriched in heavy isotopes relative to dental enamel (Δ^66^Zn_b-e_. ≈+0.2‰) (Fig. 2). The total range of isotopic variation for δ^66^Zn is 1.24‰ in dental enamel, 1.14‰ in bones and 0.70‰ in plants. In addition, carnivore values from bone (δ^66^Zn_b_ = 0.96‰ ± 0.21) and dental enamel (δ^66^Zn_e_ = 0.75‰ ± 0.24) are significantly lower than the comparable values from herbivores (δ^66^Zn_b_ = 1.40‰ ± 0.19, δ^66^Zn_e_ = 1.20‰ ± 0.21) (Fig. 3). This difference is even more pronounced when hyenas (which regularly consume bone) and crocodiles (which are the only reptiles in this assemblage) are not taken into account (Kruskal-Wallis followed by a Nemenyi test, δ^66^Zn_b_: p < 5.5 10^−6^, δ^66^Zn_e_: p < 2.3 10^−3^  Fig. 3). Some herbivore bone values exceeded the highest values observed in animals so far^3^,^13^,^14^,^20^, which is not surprising considering the small amount of data available for comparison. In comparison, browsers (δ^66^Zn_b_ = 1.34‰ ± 0. 19, δ^66^Zn_e_ = 1.11‰ ± 0. 33) are slightly higher than grazers (δ^66^Zn_b_ = 1.53‰ ± 0.23, δ^66^Zn_e_ = 1.33‰ ± 0.43) in Zn isotope values of comparable bone and dental enamel (Fig. 3). The global pattern for the whole food web is similar to one previously reported for Kruger Park, although the δ^66^Zn values from northern Kenya are significantly higher than those of Western Cape (Fig. 4, Table S4), and that all carnivore values overlap with herbivore values in the South African food webs^3^. Taken separately, Zn stable isotope values for plants and herbivores from Koobi Fora result in different distributions of values from those observed for the South African sites (Kruskal Wallis test, Table S4).

## Discussion

Although never really explained, offsets between dental enamel and bones are reported for the stable isotope ratios of all elements analyzed for these two tissues thus far (C and O^29^, Ca^6^, Mg^5^). In the present study, the offset is considerably greater for crocodiles than for mammals. Crocodile dental enamel is usually aprismatic whereas mammal enamel is prismatic^30^. If the difference in amelogenesis (enamel formation) in crocodiles versus mammals produces a different Zn isotope fractionation, this could explain the very low values in the dental enamel of the three crocodiles (Fig. 2) relative to mammals.

We also note that the difference in Zn isotope values between carnivore and herbivore mammals is less pronounced for dental enamel than for bone. The most likely explanation is linked to the fact that these tissues do not record diet during the same time period of life. Bone is renewed throughout life with a turnover of several years, while dental enamel is formed prior to adulthood without later remodeling. Bone isotopic signatures therefore correspond to the average isotopic composition of the diet while dental enamel records isotope ratios only during the period of tooth growth, which can occur over several months to a few years, depending on the mammal species and tooth type^31^,^32^. Therefore, the dental enamel values reported in this study are likely to correspond to a very short time period, depending of the age of the animal, its species, and the tooth area sampled. Depending on the sampling areas, these time periods vary between mammals, corresponding to different seasons of distinct years. Without this buffering effect of bone renewal time, distinct isotopic values associated to the consumption of specific food products can appear, especially if these specific products are consumed in a given season or time of life. As a consequence, dental enamel is likely to exhibit more various isotopic compositions than bones within a same species.

The most likely explanation for the relative differences in Zn isotope composition between the different groups of animals from Koobi Fora is related to the diet and physiology^10^,^11^. We observed significant differences between carnivore and herbivore tissue Zn isotope values, and the difference is even more pronounced when hyenas are not taken into account (Figs 2 and 3). The relative difference we report for Zn isotopic measurements of both bone and dental enamel between these animal groups is similar in magnitude to the difference previously reported for humans, between blood samples of omnivores and vegetarians^10^,^11^. In both cases, meat-consumption is associated to lower Zn isotopic ratios.

Hyenas (*Crocuta crocuta*), which are the only carnivorous mammals whose δ^66^Zn tissue values overlap with those of herbivores, regularly consume herbivore bone directly, in contrast to the other carnivores analyzed in this study^33^,^34^. As mentioned above, bones have generally higher isotopic compositions than muscle tissues^14^,^20^ although both tissues have similar Zn concentrations^35^. According to data obtained on mice, bone marrow also exhibits Zn isotopic values close to those of bone mineral^14^. Regular direct consumption of bone is then likely to significantly increase the Zn isotopic value of their diet, and thus explain the overlap in values observed for hyenas and herbivores.

Crocodiles (*Crocodylus niloticus*) also show intermediate δ^66^Zn values in bone tissue between herbivore and carnivore mammals, although not in dental enamel samples (Fig. 3). This relative placement in bone could be explained by a different fractionation between diet and bone for reptiles compared to mammals. In addition, crocodiles commonly consume foods from the aquatic ecosystem (e.g., other crocodiles and also fish), whereas we analyzed only terrestrial plants and the other animals analyzed in this study feed in the terrestrial ecosystem. As little is known concerning Zn isotopes in aquatic animals, we cannot exclude the hypothesis that their consumption could be an additional factor of variation. However, the shift between Zn isotopes composition of crocodiles and other carnivore tissues goes in the opposite direction for dental enamel and bones. Crocodile bones show higher values than other carnivore bones (Fig. 3), but their dental enamel exhibits low δ^66^Zn. Therefore, aquatic food consumption is not the best explanation for this pattern. Another possible explanation is related to the physiology of crocodiles, which frequently renew/regrow their teeth and can have irregular growth patterns. As growing animals have body tissues that are not in mass balance in terms of Zn and therefore Zn isotope ratios^36^, this may also provide an explanation of the unusual crocodile bone and enamel values. In addition, for dental enamel, depending on the time and season of tooth formation, dental enamel Zn isotope ratios may be influenced by additional fractionation due to growth or may be reflecting seasonally specific diets.

Herbivore bones from Koobi Fora are enriched in heavy isotopes relative to their diet, confirming the trend previously observed in South African food webs (Fig. 4). As expected, leaf consumption is associated with lower Zn isotopic values in bioapatite of browsers as compared with grazers.

**Figure 1:**
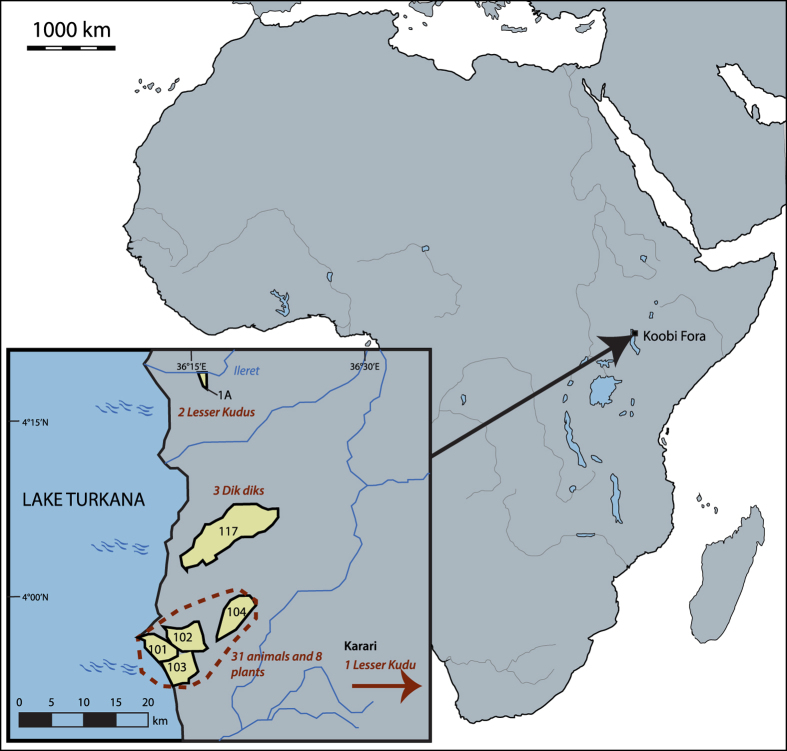
Sampling area in the Sibiloi National Park, Kenya. The areas were defined by Brown and Feibel (1986). All animals come from the areas 101, 102 and 103 except for three from the dik diks (area 117) and three from the Lesser Kudus (Two from area 1A and one from an area located outside of the map). Blank map of Africa from Daniel Dalet (histgeo.ac-aix-marseille.fr). The map showing the sampling areas was created using Adobe Illustrator CS6.

**Figure 2:**
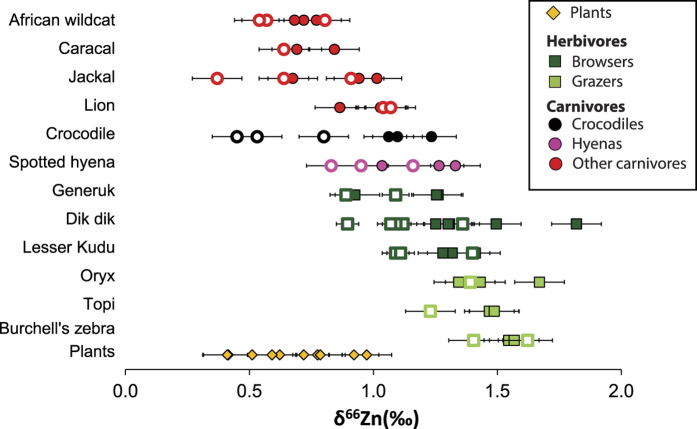
δ^66^Zn values in bones (plain symbols) and dental enamel (open symbols) grouped by species. Plant values are also displayed. Error bars represent two SD.

**Figure 3:**
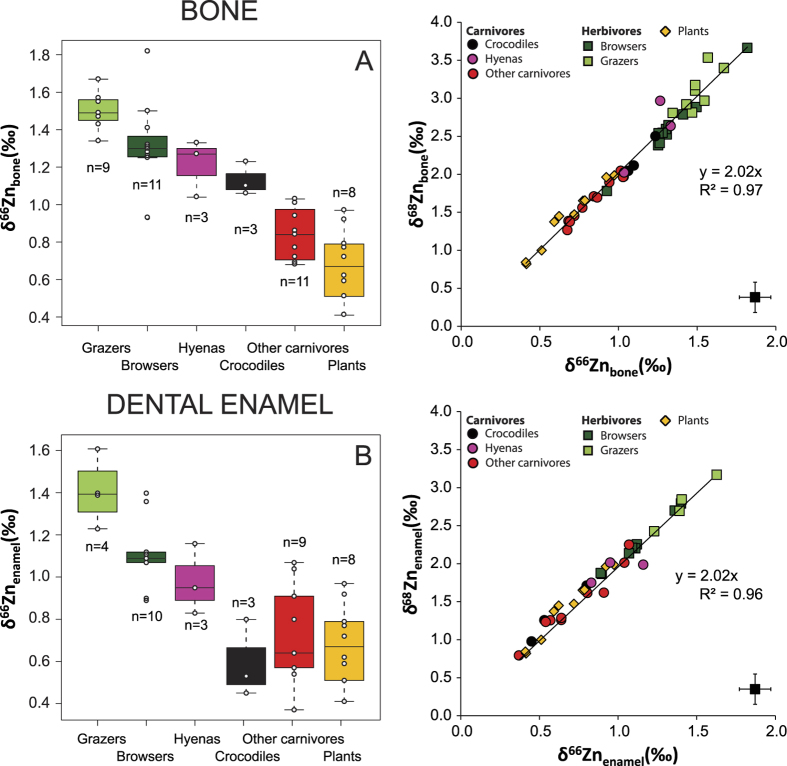
Left: δ^66^Zn range in plants as well as in bones (top, (**A**)) and in dental enamel (bottom, (**B**)) for each type of diet. The boxes represent the 25^th^–75^th^ percentiles (with the median as a bold horizontal line) and the whiskers show the 10^th^–90^th^ percentiles. Right: δ^66^Zn vs δ^68^Zn for plants and animal bones (top) and teeth (bottom) of the Koobi Fora trophic chain. As expected from mass-dependent isotope fractionation, the slopes of the least-squares straight line fit to the data are close to 2. Error bars correspond to two SD.

**Figure 4:**
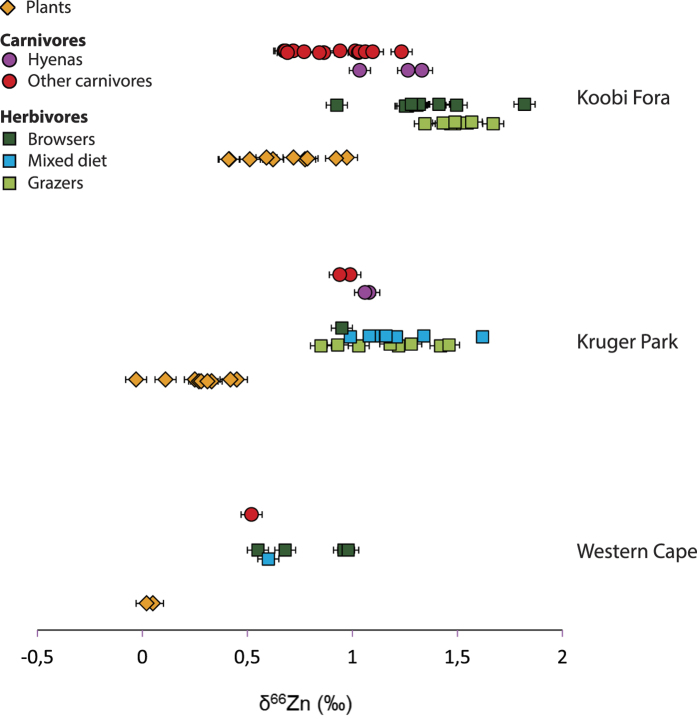
δ^66^Zn bone and plant values for three African trophic chains: Koobi Fora, Kenya (this study), Kruger Park (South Africa) and Western Cape (South Africa). Note that plant isotopic compositions vary between sites. Hyenas show higher δ^66^Zn bone values than other carnivores. Error bars corresponds to one SD.

Preliminary data for Zn isotopic variation within South African food web differ from Koobi Fora pattern, as carnivore and herbivore bone values were overlapping^36^, (Fig. 4). This overlap could be actually linked to two factors: the small sample size of the South African sites and/or the impact of the soil composition variability. Indeed, in the preliminary study of South Africa, only 4 carnivores (2 lions and 2 hyenas) of Kruger Park and one carnivore (a lion) of Western Cape were analyzed (Fig. 4). Knowing that in Koobi Fora, the lions and hyenas exhibit the highest mammal carnivore values, it can easily explain the fact that South African carnivore values all overlap with herbivore ones. In addition, different isotopic compositions of the plants at the base of the food web are likely to exist, due to geological or environmental variations – such as weathering rate or vegetation type - between sites whereas Koobi Fora areas of sampling are probably characterized by a Zn isotopic homogeneity. This hypothesis of the impact of the soil composition on the food web is supported by the different plant isotopic ranges of the three different sites (Fig. 4, Table S4). Some local variations may however exist: the dik-dik *(Madoqua guentheri)* territories usually are not bigger than 7 ha^37^ and their Zn isotopic bone values seem to depend on the specific dietary plants available in their small home ranges or the Zn isotope composition of the local soil, since values for dik-diks from the area 117 are higher than from area 102 (Fig. 1, Table S5), even if their values still fall within the browser range (Fig. 3). Further work is needed to better understand the respective influence of the underlying bedrock, diet and isotopic composition of the bioavailable Zn.

## Conclusions

The results of this study show that the Zn isotopic composition of both bone and dental enamel is strongly affected by diet. Carnivore mineralized tissues are typically depleted in the heavier Zn isotope, and are approximately 0.4–0.5‰ lower in their ^66^Zn/^64^Zn ratio compared to herbivores from the same location and food web. We showed that Zn isotopes of both bone and enamel distinguished between carnivores and herbivores within the modern Koobi Fora food web. Further work is needed to confirm if variations between types of consumers are applicable to regions with various underlying bedrock and soil Zn isotope ratio. Additionally, the preservation of dietary Zn isotope signatures in fossil teeth needs to be tested to explore the potential for palaeodietary reconstructions.

## Methods

Details of the material are given in the Supplementary Material along with additional discussion.

### Sample Collection

Bone and enamel samples come from extant animals near the Koobi Fora Base Camp, and were collected in 1984 and 1993, while plants were collected in 1986. For this study, we focused on six geographical areas as defined by Brown and Feibel^22^. The 101, 102 and 103 areas are adjacent to each other, and the 117 and 1A areas are respectively situated 20 km and 40 km north from the three other areas (Fig. 1). One lesser Kudu comes from Karari, a location situated ~30 km east from the main sampling area. The Turkana Lake represents the western limit of 101 and 103 areas. Plant samples also came from another nearby area, 104. Samples were first mechanically cleaned to remove any material potentially containing soil particles. Bone and dental enamel samples were cleaned and powdered using two different diamond drills. More details on geology are given in the Supplementary Material.

### Zinc analytical technique

Samples of bone (50 to 120 mg) and dental enamel (10 to 50 mg) were dissolved in 1 mL of double-distilled HCl 7.0 M + 0.001% H_2_O_2_. Plant samples were first digested using Supra Pur concentrated HNO_3_ (Merck) overnight, then evaporated and redissolved in HCl 7.0 M + 0.001% H_2_O_2_. Zn was purified in two steps using first the protocol of Maréchal *et al*.^27^ and secondly modified technique adapted from Moynier *et al*^38^. For this second step, the solution is evaporated to dryness and dissolved in 1 mL of HBr 1.5 M. Zn is further purified on 1 mL AG-1 × 8 resin (200–400 mesh) using 2 mL of HBr 1.5 M for matrix residue elution and 5 mL of HNO_3_ 0.5 M for Zn elution. Every preparation batch included at least one standard (in-house or reference material) and a blank. Column steps allow the quantitative recovery of the initial amount of Zn^27^,^38^. Following the protocol of Copeland *et al*.^39^ for strontium isotopes, a regression equation was used for estimating the Zn concentrations of the solution runs, based on the ^64^Zn signal intensity (V) of three solutions with known Zn concentrations (150, 300 and 600 ppb). The purified Zn fraction was measured for Zn isotopes on a Thermo Neptune Multicollector ICP-MS at the Max Planck Institute for Evolutionary Anthropology using the protocol of Toutain *et al*.^40^ and Cu doping. In-house standard Zn AA-MPI was calibrated from JMC-Lyon and used for standard bracketing. This standard corresponds to an elementary standard solution of 1000 ppm (Alfa Aesar).

All samples and standards fall on the theoretical mass fractionation line (Fig. 3). The in-house standard gave similar results to those previously reported for this standard^41^ (Table S6). Values obtained when a standard of lichen (International reference material BCR 482) was analyzed were consistent with previous analyses^18^,^42^,^43^. The Zn isotope compositions of two other reference materials (bone standard SRM 1486 and bovine liver SRM 1577c) were investigated for later use as an internal standard (Table S6). δ^66^Zn uncertainties range between 0.04‰ −0.06‰. Standard reference materials and in-house standards were analysed along with the samples. Obtained values correspond to those published elsewhere (Table S6). δ^66^Zn, δ^67^Zn and δ^68^Zn values obtained for all samples and standards measured in this study lie on a line with a slope close to the theoretical value (Fig. 1).

### Statistical tests

Our data do not follow a normal distribution, and we therefore chose to apply non-parametric tests to investigate statistical differences between taxa or groups of diet. We conducted a Kruskal-Wallis test followed by the Nemenyi post-hoc test. Results are given in the Supplementary Information (Tables S1–S4).

## Additional Information

**How to cite this article**: Jaouen, K. *et al*. Zinc isotope ratios of bones and teeth as new dietary indicators: results from a modern food web (Koobi Fora, Kenya). *Sci. Rep*. **6**, 26281; doi: 10.1038/srep26281 (2016).

## Supplementary Material

Supplementary Information
