# Supplementary material for: Zinc isotope ratios of bones and teeth as new dietary indicators: results from a modern food web (Koobi Fora, Kenya)
Source: Sci Rep. 2016 May 18;6:26281. doi: 10.1038/srep26281 (PMC4870686; doi:10.1038/srep26281)
Supplement: Supplementary Information [file srep26281-s1.pdf]

Supplementary Information

Article:

Zinc isotope ratios of bones and teeth as new dietary indicators: results from a modern food web (Koobi Fora, Kenya)

Author list

Klervia Jaouen[1], Melanie Beasley[2], Margaret Schoeninger[2], Jean-Jacques Hublin[1], Michael P. Richards[1,3]

Tables

Table S1. p values of the Kruskal Wallis tests performed on the δ^{66}Zn of bones and teeth for the different species and groups of diet. The three dietary groups are: carnivore, browser and grazer. The subgroups are "crocodiles" (non-mammal carnivores) and "hyenas" (bone-feeders).* p<0.05, **p<0.005, ***p<0.0005,** p<0.00005, *****p<0.000005**

	$\delta^{66}Zn_{bone}$	$\delta^{66}Zn_{enamel}$
Diet (without subgroups)	$1.0\ 10^{-5}$****	$1.9\ 10^{-4}$***
Diet (with subgroups)	$1.7\ 10^{-5}$****	$5.4\ 10^{-4}$**
Species	$1.4\ 10^{-3}$**	$1.8\ 10^{-2}$*

Table S2. Matrix of the p-values resulting from the Nemenyi test comparing the isotope compositions of the different types of diet (without subgroups).* p<0.05, p<0.005, ***p<0.0005, **** p<0.00005**

	Grazer		Browser	
	$\delta^{66}Zn_b$	$\delta^{66}Zn_e$	$\delta^{66}Zn_b$	$\delta^{66}Zn_e$
**Browser**	0.21	0.30	-	-
**Carnivore**	1.6e-05****	7.4 e-04****	0.007*	0.01*

Table S3. Matrix of the p-values resulting from the Nemenyi test comparing the isotope compositions of the different types of diet (with subgroups).* p<0.05, p<0.005, ***p<0.0005, **** p<0.00005**

	Grazer		Browser		Hyenas		Crocodile	
	$\delta^{66}Zn_b$	$\delta^{66}Zn_e$	$\delta^{66}Zn_b$	$\delta^{66}Zn_e$	$\delta^{66}Zn_b$	$\delta^{66}Zn_e$	$\delta^{66}Zn_b$	$\delta^{66}Zn_e$
**Browser**	0.44	0.58	-	-	-	-	-	-
**Hyena**	0.50	0.44	0.99	-	-	-	-	-
**Crocodile**	0.18	0.006*	0.81	0.07	0.99	0.49	-	-
**Carnivore**	6.3e-06****	0.0035**	0.0031**	0.047*	0.32	0.71	0.72	0.96

Table S4. Results of the Kruskal Wallis tests showing statistical differences between trophic chains for different group of animals and plants

	Kruger Park		Western Cape	
**Koobi Fora**	χ^2	p value	χ^2	p value
Plants	8.14	0.0043**	-	-
Animals	0.77	0.38	10.23	0.0014**
Carnivores	0.29	0.6	-	-
Herbivores	10.14	0.0015**	-	-
All samples	1.21	0.27	9.48	0.0021**

Table S5. Sample information (species, date and location of the collection, tooth and bone sampled) and isotopic results for Zn and N. Zn delta values are corrected for the standard JMC Lyon. Coll. Date of collection. Tooth and Bone element correspond to material sampled for Zn analyses.

								BONE				DENTAL ENAMEL			
**Id**	**Species**	**Name**	**Coll.**	**Area**	**Type**	**Tooth Element**	**Bone Element**	**$\delta^{66}Zn_b$**	**$\delta^{67}Zn_b$**	**$\delta^{68}Zn_b$**	**[Zn] ppm**	**$\delta^{66}Zn_e$**	**$\delta^{67}Zn_e$**	**$\delta^{68}Zn_e$**	**[Zn] ppm**
2340	Madoqua guentheri	Dik-dik	1984	102	Browser	P_2 to P_4	mandible	1.25	1.67	2.38	131.4	1.12	1.88	2.25	73.2
2342	Madoqua guentheri	Dik-dik	1984	102,	Browser	P^2 and P^3	maxilla	1.31	1.76	2.53	135.2	1.09	1.54	2.21	44.7
2347	Madoqua guentheri	Dik-dik	1984	102	Browser	P^3 and P^4	maxilla	1.30	1.97	2.60	117.1	0.90	1.26	1.87	81.5
4407	Tragelaphus imberbis	Lesser Kudu	1993	Ileret	Browser	M^3	maxilla	*1.29*	*2.01*	*2.59*	*116.1*				
								1.35	*2.05*	*2.71*	*127.8*				
								1.32	2.03	2.65	122.0	1.40	1.64	2.79	66.3
4438	Tragelaphus imberbis	Lesser Kudu	1993	Karari, camp to headlands	Browser	P_4	mandible	1.28	1.81	2.55	56.3	1.09	1.19	2.21	41.0
4443	Tragelaphus imberbis	Lesser Kudu	1993	Ileret, Area 1A	Browser	M^3	maxilla	*1.31*	*1.95*	*2.57*	*70.9*				
								1.51	*2.28*	*3.00*	*115.6*				
								1.41	2.11	2.79	93.2	1.11	1.16	2.20	76.0
2235	Litocranius walleri	Gerenuk	1984	101	Browser	M^1	maxilla	1.26	1.66	2.41	93.4	1.09	NA	2.21	
2170	Litocranius walleri	Gerenuk	1984	101	Browser	M^2	maxilla	1.25	1.84	2.54	82.6				
2457	Litocranius walleri	Gerenuk	1984	117	Browser	P^2	maxilla	0.93	1.38	1.78	71.1	0.89	1.43	1.88	140.8
2463	Madoqua guentheri	Dik-dik	1984	117	Browser	P^2 and P^3	maxilla	1.50	2.05	2.88	93.8	1.36	1.61	2.70	44.9
2467	Madoqua guentheri	Dik-dik	1984	117	Browser	M^1	long bone shaft fragment	1.82	2.82	3.66	61.2	1.07	1.13	2.14	49.4
2112	Crocodylus niloticus	Crocodile	1984	101	Carnivore	tooth	mandible	1.23	1.86	2.50	76.0	0.45	0.36	0.98	18.8
2098	Crocodylus niloticus	Crocodile	1984	103	Carnivore	tooth	mandible	1.06	1.55	2.05	24.0	0.53	0.88	1.26	32.6
4372	Crocodylus niloticus	Crocodile	1993	unknown	Carnivore	tooth	mandible	1.10	1.44	2.12	82.1	0.80	1.32	1.71	28.2
2139	Crocuta crocuta	Spotted Hyena	1984	101	Carnivore	premolar fragment	maxilla	1.27	2.56	2.97	111.9	0.95	1.50	2.02	65.2

								BONE				DENTAL ENAMEL			
**Id**	**Species**	**Name**	**Coll.**	**Area**	**Type**	**Tooth Element**	**Bone Element**	**$\delta^{66}Zn_b$**	**$\delta^{67}Zn_b$**	**$\delta^{68}Zn_b$**	**[Zn] ppm**	**$\delta^{66}Zn_e$**	**$\delta^{67}Zn_e$**	**$\delta^{68}Zn_e$**	**[Zn] ppm**
2230	Crocuta crocuta	Spotted Hyena	1984	101,	Carnivore	P_3	mandible	1.04	1.55	2.02	50.3	0.83	1.37	1.75	65.6
2145	Crocuta crocuta	Spotted Hyena	1984	101	Carnivore	mandibular premolar fragment	unid bone fragment	*1.30*	*1.93*	*2.62*	119.9				
								1.36	*2.03*	*2.65*					
								1.33	1.98	2.64		1.16	1.23	1.99	49.1
2197	Canis sp.	Jackal	1984	103	Carnivore	M^2	parietal	0.68	0.88	1.27	155.1	0.37	0.49	0.79	73.8
2191	Canis sp.	Jackal	1984	101	Carnivore	P^4	zygomatic	1.01	1.57	2.05	132.1	0.91	1.02	1.62	49.1
2241	Canis sp.	Jackal	1984	101	Carnivore	M^2	parietal	0.94	1.44	1.90	126.4	0.64	0.88	1.26	132.9
2192	Felis silvestris	African wildcat	1984	101	Carnivore	P^3 and C^1	zygomatic	0.72	1.03	1.45	144.6	0.57	0.99	1.25	74.6
2203	Felis silvestris	African wildcat	1984	101	Carnivore	M_1	skull and mandible	0.68	1.01	1.39	173.7	0.54	0.81	1.23	94.4
2448	Felis silvestris	African wildcat	1984	117	Carnivore	M_1	maxilla	0.77	1.10	1.56	107.7	0.80	1.18	1.62	120.7
2436	Caracal caracal	Caracal	1984	117	Carnivore	no tooth	radius	0.84	1.37	1.71	93.0				
2416	Caracal caracal	Caracal	1984	unknown	Carnivore	M_1	mandible	0.69	0.93	1.38	110.2	0.64	0.94	1.29	74.7
2445	Felis leo	Lion	1984	117	Carnivore	P_4	mandible	1.03	1.30	1.97	150.5	1.04	1.47	2.01	167.4
2426	Felis leo	Lion	1984	unknown	Carnivore	C	mandible	1.03	1.31	1.96	139.9	1.07	1.67	2.25	71.2
2425	Felis leo	Lion	1984	unknown	Carnivore	no tooth	zygomatic	0.86	1.19	1.69	67.8				
2085	Damaliscus (lunatus) korrigum	Topi	1984	101,	Grazer	M^1	long bone shaft fragment	1.49	2.42	3.10	76.9				

								BONE				DENTAL ENAMEL			
**Id**	**Species**	**Name**	**Coll.**	**Location**	**Type**	**Tooth Element**	**Bone Element**	**$\delta^{66}Zn_b$**	**$\delta^{67}Zn_b$**	**$\delta^{68}Zn_b$**	**[Zn] ppm**	**$\delta^{66}Zn_e$**	**$\delta^{67}Zn_e$**	**$\delta^{68}Zn_e$**	**[Zn] ppm**
2094	Damaliscus (lunatus) korrigum	Topi	1984	103	Grazer	P_3	mandible	1.47	1.96	2.81	94.7	1.23	1.82	2.43	53.4
2175	Damaliscus (lunatus) korrigum	Topi	1984	101	Grazer	M_2	mandible	1.49	2.52	3.17	59.0				
2120	Oryx beisa	Oryx	1984	102	Grazer	P_2	mandible	1.34	2.26	2.81	80.7				
2131	Oryx beisa	Oryx	1984	102	Grazer	M_1	mandible	*1.62*	*2.47*	*3.23*	*79.8*				
								1.72	*2.78*	*3.56*	*64.0*				
								1.67	2.62	3.40	71.9				
2132	Oryx beisa	Oryx	1984	102	Grazer	P^4	maxilla	1.43	1.99	2.92	60.3	1.39	2.02	2.69	14.1
2142	Equus burchelli	Burchell's Zebra	1984	101	Grazer	M_2	no bone					1.61	2.30	3.13	130.6
2144	Equus burchelli	Burchell's Zebra	1984	btw petrol station and camp	Grazer	molar	femur	1.55	2.11	2.97	106.7				
2306	Equus burchelli	Burchell's Zebra	1984	102	Grazer	molar	unid bone fragment	1.57	2.94	3.53	69.2	1.40	2.06	2.85	34.9
3234		Acacia	1986	102	Plant			0.77	1.03	1.65	6.8				
4381		Acacia pods	1993	102	Plant			0.41	0.53	0.82	8.3				
3245		Bush	1986	103	Plant			0.41	0.53	0.84	9.4				
3239		Grass	1986	near lake (Galana Bori)	Plant			0.51	0.61	1.00	13.1				
3241		Grass	1986	104	Plant			0.62	1.19	1.45	8.9				
3243		Grass	1986	104	Plant			0.97	1.33	1.99	6.4				
3244		Grass	1986	103	Plant			0.59	0.70	1.37	8.6				
3231		Legume	1986	102	Plant			0.72	1.05	1.47	28.4				
3246		Succulant	1986	103	Plant			0.79	1.16	1.66	2.5				
3232		Vine	1986	102	Plant			0.92	1.52	1.96	10.3				

Table S6. $\delta^{66}Zn$ values of in house standard and reference materials. Zn delta values are corrected for the standard JMC Lyon

	Category	Material	n	$\delta^{66}Zn$	SD	Expected value	Reference
**AZE**	in house standard	bone	8	1.47‰	0.11	1.50 ‰ ± 0.04	Jaouen (2012)
**SRM 1486**	reference material	bone meal	6	1.17‰	0.07	never measured	
**SRM 1577c**	reference material	bovine liver	4	-0.13‰	0.02	never measured	
**BCR 482**	reference material	lichen	7	0.03‰	0.08	0.07‰ ± 0.1	Cloquet et al., 2006a, 2006b
						0.14‰ ± 0.03	Viers et al. 2007
						0.09‰ ± 0.04	Sonke et al. 2008

Table S7. Descriptive statistics for Zn isotopes in bones and dental enamel.

	$\delta^{66}Zn_{bone}$	$\delta^{66}Zn_{enamel}$
N	36	29
Min	0.68	0.37
Mean	1.26	0.97
Max	1.82	1.61
SD	0.30	0.32

Table S8 R^2 and p values for linear regression between $\delta^{66}Zn$ and light element isotopic delta as well as Zn content

**BONE**	**$\delta^{66}Zn_{bone}$**	
	R^2	p
Zn content	0.1759	0.019*
**DENTAL ENAMEL**	**$\delta^{66}Zn_{enamel}$**	
	R^2	p
Zn content	0.0035	0.76

Additional information

Geological context: The bedrock of the Koobi Fora region is characterized by a sequence of tuffs, which contain around 3 % of Fe and 200 ppm of Zn (Brown and Fleibel, 1986). Locations of the different areas where the samples were collecting is given in Brown and Fleibel (1996) and the localities in the table S5**.**

Additional discussion

Zn content as dietary indicator:

According to data previously reported, Zn content is higher in mammal carnivore tissues than herbivore ones, when hyenas are not taking into account (Figure S1). However, the difference is much lower than the one previously reported in dental enamel (Kohn et al., 2013), and the one observed for Zn isotopes in this study. As these concentrations were not precisely measured but simply estimated with Zn signal during isotope analyses, it is possible we lost part of the biogenic signature. Still, given that Zn concentrations are regulated by the body which keeps them under homeostatic conditions (Cousins et al., 1986), we believe that their isotopic composition is more likely to trace the animal diet.

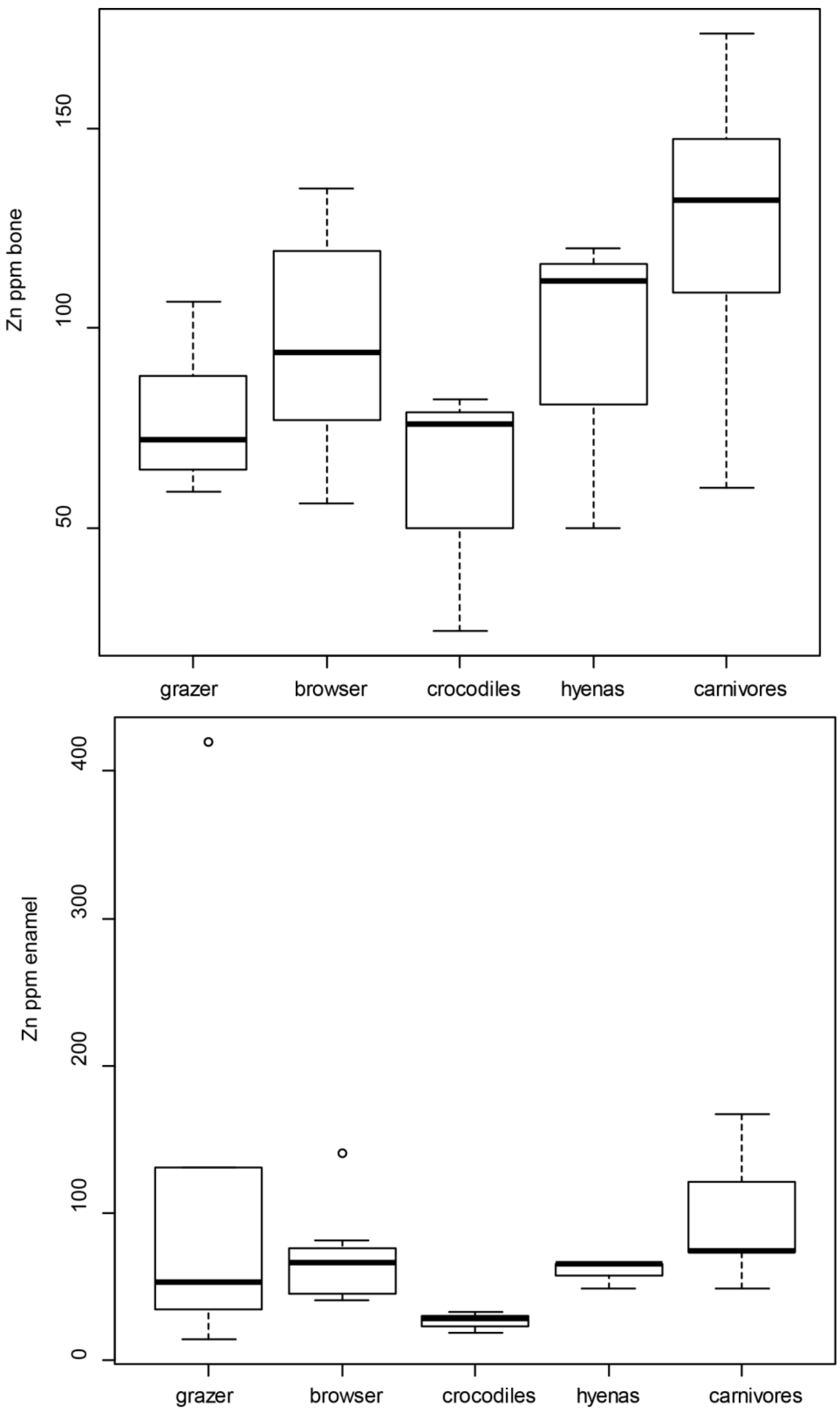

Figure S1. Zn content of bones (on the top) and dental enamel for grazers, browsers, crocodiles, hyenas and other carnivores of the Koobi Fora trophic chain.
